# CRISPR-Cas Technology as a Revolutionary Genome Editing tool: Mechanisms and Biomedical Applications

**DOI:** 10.61186/ibj.27.5.219

**Published:** 2023-06-18

**Authors:** Sahar Ebrahimi, Mohammad Ali Khosravi, Abbasali Raz, Morteza Karimipoor, Parviz Parvizi

**Affiliations:** 1 Molecular Systematics Laboratory, Parasitology Department, Pasteur Institute of Iran, Tehran, Iran;; 2 Molecular Medicine Department, Biotechnology Research Center (BRC), Pasteur Institute of Iran, Tehran, Iran;; 3 Malaria and Vector Research Group (MVRG), Biotechnology Research Center (BRC), Pasteur Institute of Iran, Tehran, Iran

**Keywords:** CRISPR-Cas system, Genome editing, Medicine, Parasitology

## Abstract

Programmable nucleases are powerful genomic tools for precise genome editing. These tools precisely recognize, remove, or change DNA at a defined site, thereby stimulating cellular DNA repair pathways that can cause mutations or accurate replacement or deletion/insertion of a sequence. The CRISPR-Cas9 system is the most potent and useful genome editing technique adapted from the immune system of certain bacteria and archaea against viruses and phages. In the past decade, this technology has made notable progress, and at present, it has largely been used in genome manipulation to make precise gene editing in plants, animals, and human cells. In this review, we aimed to explain the basic principles, mechanisms of action, and applications of this system in different areas of medicine, with an emphasize on the detection and treatment of parasitic diseases.

## INTRODUCTION

**Fig. 1:**
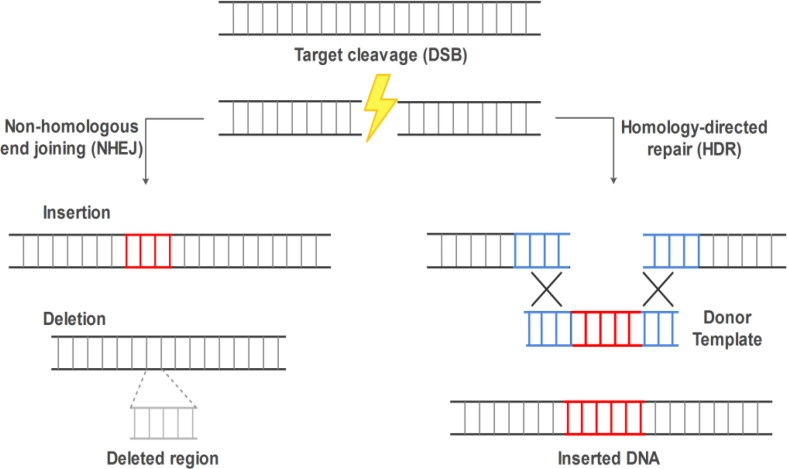
CRISPR/Cas9-mediated DSB repair mechanism. The Cas9 introduces a DSB in the target DNA. DNA repairing includes the NHEJ and HDR pathways. The NHEJ pathway leads to Indel. The HDR pathway uses homologous donor DNA sequences to create precise insertion between DSB sites

Genome editing technology is a powerful tool for manipulating the genome, which allows scientists make highly specific changes in the DNA sequence of living organisms, including plants, bacteria, and animals. Gene editing is performed using programmable nucleases to target a specific DNA sequence, in which DNA is inserted, removed, or modified[1-3]. Genome editing nucleases induce targeted DSBs. DSBs are subsequently repaired by cellular mechanisms via NHEJ in the absence of a donor template or HDR in the presence of a donor template. HDR can be used for gene integration or base correction, while NHEJ is used to create Indels, which causes disruption of the target gene[4] (Fig. 1). Gene editing technology has been emerged in the 1990s, and three site-specific genome editing tools, namely ZFN, TALEN, and CRISPR-Cas9, each with its pros and cons, have widely been used (Table 1). The inspiration of the natural zinc fingers leads to the development of the first genome editing tool, ZFN, which is a fusion of a customizable DNA-binding protein and *Fok I* endonuclease to create DSB[5-7]. The discovery of transcription activator-like effectors in late 2009 resulted in the consideration of the TALEN as an alternative platform to ZFNs as engineering programmable DNA-binding proteins. TALEN is also comprised of DNA-binding and *Fok I* cleavage domains. In comparison with the ZFN, TALEN benefits from two important advantages: first, lower toxicity and higher specificity, and second, the simpler design[8,9]. Genome editing technology has taken a leap forward since the discovery of CRISPR-Cas in 1987[10,11].

In comparison with ZFNs and TALENs, CRISPR/ Cas9 is not only a versatile, robust, and highly precise genome editing tool, but also is very quick, inexpensive, and extremely simple to use. Unlike ZFN and TALEN that recruit proteins to recognize the targeted sequences in the genomic regions, the CRISPR/Cas system uses a crRNA to recognize the targeted genomic sequence and acts as a scaffold for recruiting the Cas endonuclease to introduce site-specific DSBs[12]. This review provides background knowledge on the history and mechanism of CRISPR/Cas systems, the classification of CRISPR-Cas systems, and the application of CRISPR as a genome editing tool. The application of CRISPR systems has been discussed for treatment and diagnosis, with more focus on infectious diseases and parasites. 

**Table 1:** Comparison of the features of three gene editing techniques^[^^13^^-^^15^^]^.

**Genome editing tool**	**ZFN**	**TALEN**	**CRISPR-Cas9**
Component	ZFP + FokI Fusion protein	TALE + FokI Fusion protein	gRNA + Cas9 Protein
Nuclease	FokI	FokI	Cas9
DNA-binding domain	Protein	Protein	RNA
Target site size	9-18 bp	30-40 bp	22 bp + PAM sequence
Design availability	More complex	Complex	Very simple
Ease of multiplexing	Low	Low	High
Time	7-10 days	5-7 days	1-3 days
Cost	High	High	Low
Ex vivo delivery	Easy	Easy	Easy
Efficiency	Variable	High	High
Methylation sensitivity	High	High	Low

***CRISPR/Cas9 System***

*A brief history of the CRISPR/Cas9 system*

CRISPR/Cas systems are repeating DNA sequences in bacteria and archaea and act as adaptive immune response systems that protect the mentioned organisms against invading viruses, such as bacteriophages and plasmids. In 1987, CRISPR systems were first discovered in the *Escherichia coli *genome by a Japanese scientist, Yoshizumi Ishino. This system comprises a set of 29 nucleotide repeats interspaced by five intervening 32-nucleotide non-repetitive sequences in the *Escherichia coli *genome[16]. In 1993, CRISPR clustered repeats were discovered in *Mycobacterium tuberculosis* by van Embden[17] and his team who identified different spacer sequences between the DNA repeats. This clustered array was highly conserved across multiple evolutionary distinct bacterial genomes, and the locus was named CRISPR by Francisco Mojica, a microbiologist at the University of Alicante in Spain, in 2002[18]. In 2010, the CRISPR/Cas system of *Streptococcus thermophilus* was shown to introduce DSBs at a precise position in the target DNA[19]. In 2011, Deltcheva and colleagues[20] reported that tracrRNA, CRISPR-Cas9, and RNase III are essential for maturation of crRNAs in *Streptococcus pyogenes*. One year later, in 2012, it was discovered that the CRISPR system of *Streptococcus pyogenes* serves as a genome editing tool by showing that the Cas9 can be guided by tracrRNA, crRNA, and a synthetic sgRNA to cleave target DNA in vitro[10^,^^21^]. Since then, the CRISPR/Cas9 technology has been applied for targeted and precise manipulations of DNA in various cell types and organisms[22-26].

*Components of CRISPR/Cas9 system*

According to the structure and function of the effector complex (Cas proteins), the CRISPR/Cas system is classified into two main classes with six types. Class I comprises types I, III, and IV. Class II system comprises types II, V and VI. The first and most common type used in genome editing is the type II CRISPR/Cas9 system, which includes three main components: Cas9 protein, crRNA, and tracrRNA. The effector protein of class I consists of a multiprotein complex, while the class II system utilizes the Cas9 protein, which is a single, large, and multidomain Cas protein[27,28]. As an RNA-guided endonuclease, SpCas9 has been adapted for targeted genome editing in a variety of organisms. Cas9 contains two parts: a recognition domain, which is responsible for binding gRNA to target nucleic acid, and a NUC domain, which includes HNH, RuvC lobes, and PAM-interacting domains. In the NUC, the RuvC-like nuclease cleaves the non-complementary (non-target) DNA strand, and the HNH cleaves complementary (target) DNA strand. The PAM-interacting domain is responsible for identifying the PAM sequence and binding to the non-complementary strand of the target DNA[29-31].

*Mechanisms of CRISPR/Cas9-based genome editing*

The critical part of the CRISPR/Cas9 tool is sgRNA, which is a combination of crRNA and tracrRNA. The crRNA is a 20-bp sequence complementary to the target DNA site (also called protospacer), located at the 5′ end of sgRNA, and tracrRNA serves as a binding scaffold for the Cas9 endonuclease. Easily programmable sgRNA can effectively recognize specific sequences and guide the Cas9 endonuclease to the target site by simple Watson–Crick base pairing and makes DSBs 3 or 4-bp upstream of the PAM sequence at a target site[10]. The specific binding of CRISPR/cas9, besides the 20 bp complementarity sequence of gRNA, requires a PAM sequence, i.e. a short and conserved sequence of 2-5 bp length located next to the target site. The PAM sequence and its size depend on the bacterial species. The PAM sequence for *Streptococcus pyogenes* Cas9 is 5ʹ-NGG-3ʹ[21^-^27,32-35], for *Streptococcus thermophiles* is 5'-NGGN G-3' or 5'-NNAGAAW-3'[36^,^^37^], for *Staphylococcus aureus* is 5'-NNGRRT-3' or 5'-NNGRR(N)-3'[38,^39^], for *Neisseria meningitidis* is 5'-NNNRRT-3' or 5'-NNNNGMTT-3'[^40^], and for *Francisella novicida* (FnCpf1) is 5'-NGG-3'[^41^]. Nucleotide bases are represented by N; G shows guanine, R indicates purine A or G, M represents nucleotide A or C, and W denotes weak bond A or T.

***Different Cas types***

**Fig. 2:**
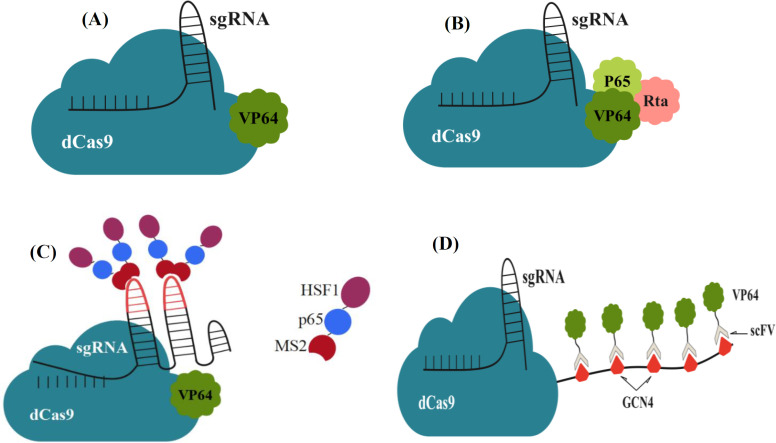
Gene activation by engineered CRISPR systems. (A) The dCas9-VP64 was generated by fusing dCas9 with VP64, a strong transcriptional activator domain (four tandem copies of VP16, herpes simplex viral protein 16). (B) The VPR system is dCas9-VP64, which is fused with two other strong transcriptional activators (p65 and Rta). The VPR system is stronger than the dCas9-VP64 in gene activation. (C) The SAM system is a dCas9-VP64 with engineered sgRNAs fused with MS2, HSF1, and p65 proteins, which shows the highest levels of gene activation. (D) The SunTag system comprises dCas9 fused to tandem GCN4 peptide repeats, a single chain variable fragment (scFv) antibody, and multiple copies of the VP64

In order to enhance precise genome editing using the CRISPR-Cas system, several other Cas endonuclease proteins and Cas9 variants with different features and PAM specificities have been studied and developed as gene editing tools. Three variants of the Cas9 as genome editors have been designed and used so far. The first, WT Cas9, cleaves double-stranded DNA at a target site using HNH and RuvC-like NUCs[^42^^-^^45^]. Various methods have been developed to increase on-target efficiency and reduce potential off-target mutagenesis of WT Cas9 such as shortened and modified sgRNAs, purified Cas9 ribonucleoproteins, engineered Cas9 protein, *FokI*-Cas9 fusion nucleases, and paired catalytic mutant Cas9n. WT Cas9 can be converted into second variant Cas9n by introducing point mutations in one of the NUCs (RuvC^D10A^ or HNH^H840A^). Cas9n produces single-strand breaks rather than a DSB. To produce DSBs with Cas9n, specific binding of two gRNAs, placed on opposite strands, is required[^46^^-^^48^]. Cas9^D10A^ nickase was successfully exploited to target genes for the DNA-damage response proteins MDC1, 53BP1, RIF1 and P53, and Lamin A/C (the nuclear architecture proteins) in three different human cell lines[^45^]. The third variant of SpCas9 is nuclease-deactivated Cas9, called dCas9, which is produced through D10A and H840A mutations at the HNH and RuvC NUCs, respectively. The dCas-9 nuclease does not have DNA cleavage activity, but its DNA-binding activity has been still retained[^49^]. Thus, the dCas9 can be utilized to precisely and specifically bind to the targeted site within the genome, without cutting the DNA. The CRISPR/dCas9 DNA-targeting technique has various applications in many areas. For instance, the dCas9 protein can be fused with a variety of functionally active domains to directly modify transcription without genetically changing the DNA sequence[50^-^^52^]. Effector domains may include epigenetic repressing domains to create CRISPR inhibitors, such as Kr*ü*ppel-associated Box or Sin3a-interacting domain, that silence the expression of the target gene by interfering with transcriptional initiation (via obstruction of RNA polymerase binding and elongation[53^-^^55^]. The CRISPR/dCas9 can also be fused to the transcription activator domains to produce a CRISPR activator for gene activation by recruiting transcription factors to the target gene. Until date, many transcriptional activators have been developed. VP64 (4× fusion of the VP16 transcriptional activation protein derived from herpes simplex virus) is a widely used activator domain for modest gene activation[^56^^-^^59^] (Fig. 2A). VPR is a tripartite complex consist of VP64, P65, and Rta fused with dCas9 in order to activate transcription[^60^] (Fig. 2B). SAM is a combination of dCas9/VP64 protein engineered with aptamers, which binds to MS2, HSF1, and p65 proteins. dCas9-VP64-SAM acts as a strong transcriptional activator[61^,^^62^] (Fig. 2C). SunTag system uses up to 24 repeats of VP64 instead of a single copy at each target site. Thus, dCas9-SunTag recruits more transcriptional machinery to the targeted gene. The limitation of this platform is the complexity of its construction[63,64] (Fig. 2D). Epigenetic modifications are a wide range of changes in gene expression without altering the DNA sequence. Epigenetic dysregulation is correlated with alterations in gene expression levels and disease states. Recently, targeted editing of the epigenome has become feasible using the CRISPR/Cas9 system. The dCas9-TET1 system is a dCas9 fused with the TET1 protein that is employed for DNA demethylation by oxidizing 5-methylcytosine to 5-hydroxymethylcytosine. Targeted DNA methylation at the specific site is achievable by dCas9-DNA methyltransferase[^65^-^67^].

*CRISPR-Cas12a (Cpf1)*

CRISPR-SpCas9 from *Streptococcus pyogenes* is type II, subtype II-A of CRISPR/Cas Class 2 systems, which is the first and most popular genome editing tool. Class II type V is divided into four subtypes (V-A, V-B, V-C, and V-U). The CRISPR-Cas12a from *Prevotella* and *Francisella 1* bacteria belongs to class II, type V, and subtype V-A CRISPR system. Cas12a is an endonuclease enzyme, which comprises ∼1,300 amino acids, and it is a little smaller than SpCas9 with 1,368 amino acids. There are some differences between Cas12a and Cas9 proteins. Cas12a, in contrast to Cas9, does not require a tracrRNA for the biogenesis of mature crRNA. Unlike Cas9, Cas12a recognizes 5' T-rich PAM sequences "TTN/TTTN/TTTV" (N=A, T, C, G; V=A, C, G) to generate DSBs. Cas12a cleaves targeted sequence, 18-23 nt in the downstream of the PAM in a staggered pattern (4 or 5 nt overhang), to create sticky ends, contrary to blunt ends produced by Cas9. Cas9 possesses two endonuclease domains (RuvC and HNH), 

while Cas12a lacks the HNH NUC[^68^^-^^73^]. Because Cas12a has ssDNA cleavage activity, it can be employed for nucleic acid detection and genome manipulation. Chen et al.^ [^^74^^]^ combined the LbCas12a with RPA, a sensitive and selective isothermal amplification technique, to develop a rapid method known as DETECTR. This method has been exploited for nucleic acid detection of two high-risk types of HPV 16 and 18 with attomolar sensitivity. In this study, the extracted viral DNA was amplified by isothermal amplification. crRNAs are designed to target the hypervariable loop V region of the L1 gene of HPV16 and 18. The crRNA and target DNA binding trigger LbCas12a proteins to cleave the target and nontarget DNA in which fluorophores and quenchers are connected to different sides of ssDNA[^74^].

*CRISPR-Cas13a*

CRISPR-Cas13a, previously known as CRISPR-C2c2, from *Leptotrichia* species, belongs to the type VI class II CRISPR-Cas system. CRISPR-Cas13a is the most recently identified editing tool with RNA-guided ribonuclease activity, which can be programmed to cut single-stranded RNA molecules. The CRISPR-Cas13a system contains an effector protein with two predicted conserved higher eukaryotes and prokaryotes nucleotide-binding RNase domains, which are responsible for preferentially cutting the targeted RNA at uracils. Previous studies have shown that Cas13a is an applicable and effective protein for cutting and editing of targeted RNA in both prokaryotic and eukaryotic cells, as well as nucleic acids detection with high sensitivity[^75^^-^^80^]. In 2017, Gootenberg et al.[^75^] developed a novel CRISPR/Cas13a-based nucleic acid detection platform called SHERLOCK, which is created by combining the collateral cleavage effect of Cas13a with T7 transcription and RPA for detection of Zika and dengue viruses with attomolar sensitivity and specificity. The RPA-SHERLOCK platform was also used by Li et al.^[^^81^^] ^to detect the RNA-based CCHFV, a severe hemorrhagic fever virus. Within 30-40 minutes, the CCHFV was identified using a rapid, sensitive, and precise method with a limit of detection of 1 copy/μl. In a different study published in 2019, Liu et al.[82] used the SHERLOCK platform (CRISPR/Cas13a, RT-RPA, and T7 transcription) to detect Avian Influenza A (H7N9) virus nucleic acid by targeting its HA and NA genes. The platform successfully detected one femtomole of HA and NA within 50 minutes. 

*CRISPR-Cas14*

The newly discovered Cas14, with 400 to 700 amino acids, two folds shorter than Cas9 (~1400 amino acids), is the smallest and most compact RNA-guided nuclease recognized till now. Cas14 was found in the DPANN, a superphylum of archaea that contains the smallest cell and genome size compared to other archaea. Cas14 binds to ssDNA and cuts it without requiring a PAM flanking the target DNA sequence. The non-specific ssDNA trans-cleavage activity of Cas14 has enabled more efficient and robust applications of this system in the development of DNA detection platforms, such as diagnosis of microbial infections, ssDNA viruses, and detection/modification of cancer cells[^83^^-^^85^].

*CRISPR-Cas12b*

CRISPR-Cas12b (C2c1) is an intriguing genetic engineering tool/genome editing system, which changes the molecular biology landscape. It is an RNA-guided nuclease derived from the Type II CRISPR-Cas bacterial adaptive immune system. Discovered in the early 2000s, the tool has revolutionized the development and application of genome editing technology. At the structural level, Cas12b is a 20-25 kDa single polypeptide that consists of two separate domains: the RuvC and HNH. The RuvC domain is responsible for unwinding the targeted DNA sequence, while the HNH domain performs the cutting action. Working in concert, the two domains can quickly identify a specific DNA sequence and cut it out to convert a single base pair mutation. In terms of clinical application, Cas12b has been repurposed to enable mammalian genome editing. Numerous studies have demonstrated its ability to introduce the desired mutations at the single base pair resolution in the genomes of living mammals. In addition to genome editing, the versatile nature of Cas12b can also be used to detect viruses at ultrasensitive scales. By employing the C2c1-based diagnostic system, the detection of viruses can be achieved with high accuracy, specificity, and speed. Most recently, the Cas12b platform has been used to develop genetic circuitries via combinatorial assembly and engineering. This progress paves the way for the development of new approaches to gene therapy, gene manipulation, and the creation of designer organisms[^86^^-^^89^]. As the body of knowledge surrounding C2c1 continues to expand, the opportunities for broadening its uses increase. By its broadening scope and its efficient and precise delivery of genetic modifications, it is no wonder that Cas12b is now a central figure in modern molecular biology and genetics. This ongoing progress of the Cas12b technology promises to revolutionize the biomedical industry at the cutting edge of genome engineering.

*CRISPR-Cas7-11*

The CRISPR-Cas7-11 (CRISPR-Cas III-E) is an advanced type of CRISPR-Cas technology capable of programmable RNA targeting with a single-protein effector. CRISPR-Cas7-11 is unique in its design and utility compared to other CRISPR-Cas systems and has been the subject of intense study to further understand its structure and underlying engineering. The Cas7-11 protein is a single molecule of a complex design composed of three subunits: a CRISPR-associated protein, a subunit of structure (CSE), and an RBD. The core of the protein is the CSE, which forms an RNA-binding platform for gRNA molecules. An RBD, composes of four distinct alpha-helical domains, interacts with the CSE and the gRNA to form a platform for executing programmable RNA cleavage. The CRISPR-Cas7-11 system works by targeting foreign RNA molecules. The gRNA attached to the CSE of the Cas7-11 protein is programmed to recognize an mRNA molecule of interest. Once the mRNA is identified, the RBD inflates and interacts with the target RNA, leading to its cleavage. This action results in an mRNA degradation pathway, with the cleavage of the targeted mRNA molecules precluding translation of the sequence and resulting in the loss of expression of the target protein. The unique architecture of Cas7-11 and its programmable RNA targeting ability have enabled it to be utilized in applications related to RNA manipulation and gene editing^[^^90^^,^^91^^]^. The Cas7-11 system has already been used in research on the issues relating to transcriptome inhibition, target gene expression manipulation, and RNAi in a single-protein system. Further development of the protein has enabled more efficient localization of the various CRISPR-associated RNAs at different sites and further optimized the system for target genes and mRNA editing. The CRISPR-Cas7-11 technology has the potential to revolutionize gene editing and RNA manipulation due to its efficient and single-protein design. With further study of the CRISPR-Cas7-11 system, scientists may be able to develop new tools and techniques for precision genetic engineering, disease treatment, and even synthetic biology. One of the unique features of the CRISPR-Cas7-11 system is the use of a single protein, known as Cas7, to target and cut specific RNA molecules. This view is in contrast to other CRISPR systems, such as CRISPR-Cas9, which requires multiple proteins and a gRNA to achieve the same function. The simplicity of the CRISPR-Cas7-11 system makes it easier to engineer and use in a wide range of applications. Another advantage of the CRISPR-Cas7-11 system is its ability to target RNA molecules that are not translated into proteins. These include regulatory RNAs that play an important role in gene expression and cell signaling. By manipulating these non-coding RNAs, scientists could develop new therapies for diseases that are difficult to treat using traditional gene editing techniques. However, there are still many challenges to be overcome before that the full potential of the CRISPR-Cas7-11 system can be realized. These challenges include improving the specificity and accuracy of the system to minimize off-target effects, as well as developing delivery methods that can efficiently introduce the system into cells and tissues[^92^]. Despite these challenges, the CRISPR-Cas7-11 system represents a promising avenue for genetic engineering and RNA manipulation that could have far-reaching implications for medicine, industry, and beyond.

***Different types of Cas proteins***

By comparing and contrasting the different Cas proteins, researchers can gain a better understanding of their individual functions, and how they work together. This knowledge can then be used to develop new applications for CRISPR-Cas technology, which has the potential to revolutionize genetic engineering. It can also help scientists explore new treatments for genetic diseases and create more effective therapies. Each type of Cas protein has a different function and structure. For instance, Cas9 proteins have a DNA cutting function, while Cas12a proteins possess a DNA cleaving function[^34^^,^^69^^,^^78^^,^^85^^,^^93^^,^^94^] (Table 2).

**Applications of CRISPR-Cas Systems**

In just ten years, the RNA-programmable site-specific CRISPR–Cas genome editing tool has become one of the most famous discoveries in biology and has an enormous impact on the life sciences. Scientists have applied CRISPR systems in many areas, including the medical field, biotechnology, and agriculture. Herein, we review some of the main applications of CRISPR technology.

***Establishing cell and animal models of human diseases***

The development of cellular and animal models is one of the early applications of the CRISPR-Cas system, which is useful to realize the reason behind diverse diseases and clarify molecular pathways used for more effective therapeutic strategies. Traditional methods for generation of cell and animal models of human diseases are complex, costly and time-consuming. Since the discovery of CRISPR-Cas systems, the generation of genetically modified cell and animal models has become significantly simpler in a highly efficient approach. Genetically modified animal models created using CRISPR technology, have a wide range of applications, such as their use in pharmaceutical and biotechnological production. They can also be used to study specific genes, investigate their functions and evaluate the mechanisms and progression of diseases in order to assess potential therapies. As one of the most common models for biological research, as well as animal studies (rats, Caenorhabditis elegans, drosophila, zebrafish, frogs, pigs, goats, and cynomolgus monkey models), the CRISPR-Cas tool has already been used to manipulate genes in mice[^95^^-^^100^]. So far, modeling diseases with CRISPR have been created for lung, pancreatic, brain, and hematopoietic cancers[^101^^-^^107^], cardiovascular disorders, cardiomyopathy[108], muscular dystrophy[^109^], HD[^110^], albinism[^111^], obesity[^112^], hemophilia B[^113^], and infectious diseases. In addition, the CRISPR-Cas system has been used to produce in vitro models for a wide variety of diseases. CRISPR-Cas can edit multiple sites, simultaneously, by delivery of several gRNAs and Cas endonuclease using different strategies (Table 3). This unique advantage makes this tool an applicable technique for producing a cancer model with complexity similar to that occurs in humans. Matano et al.^[^^114^^]^ generated a colorectal cancer model by targeting multiple genes in the human colon epithelium using the CRISPR-Cas9 genome-editing tool. Heckl et al.^[^^104^^]^ generated a model of AML by using the CRISPR-Cas9 to introduce multiple gene mutations, including epigenetic modifiers, transcription factors, and cytokine signaling genes in mouse HSCs. The iPSCs are highly attractive cell resources in disease modeling and regenerative medicine based on their unlimited self-renewal and multiple differentiation capability. In recent years, numerous novel disease models have been generated by CRISPR-Cas9-edited iPSCs, including Parkinson’s, Niemann-Pick type C, and SCD, as well as β-thalassemia, Rett syndrome, cystic fibrosis, and α1-antitrypsin deficiency[115^-^^117^]. 

**Table 2:** Comparison of different types of Cas proteins and their characterization

**Cas protein**	**Source**	**Amino acid size**	**Protein size** **(kDa)**	**PAM sequence**	**Cutting ** **site**	**Nucleic acid ** **cleavage**
Cas9	*Streptococcus pyogenes*	1,368	10-14	NGG	~3 bp 5′ of PAM	DNA blunt ends
						
Cas12a	*Acidaminococcus* sp.	1,348	7-8	TTTV	~12 bp 5′ of PAM	DNA staggered end
						
Cas12b	*Lachnospiraceae bacterium* ND2006	1,365	6-7	TTTN	~3 bp 5′ of PAM	DNA blunt ends
						
Cas13a	*Leptotrichia shahii*	1,204	8-9	GNNG	~4 bp 5′ of PAM	DNA staggered end
						
Cas13b	*Lachnospiraceae bacterium* A2-165	1,138	8-9	GNNN	~4 bp 5′ of PAM	DNA blunt ends
						
Cas14	*Neisseria meningitidis*	1,204	2-3	ATTT	~4 bp 5′ of PAM	DNA staggered end

**Table 3:** Common CRISPR/Cas9 delivery strategies

**Methods**			**Delivery format**	**Efficiency**	**Safety**	**Cost**	**Advantages**	**Disadvantages**
**Delivery strategies**	**Chemical and physical delivery methods**	Lipid nanoparticles	DNA, mRNA protein	Low	Low	Low	FDA-approved; Low stress to the cells	Requires extensive optimization, variable efficiency
						
		Electroporation	DNA, mRNA protein	High	Low	High	Easy to operate	Cell viability issue; in vivo work difficult
						
		Microinjection	DNA, mRNA protein	Low	Low	High	Direct delivery; Dosage more controllable	Technical challenging; in vivo work not feasible
						
		Cell penetrating peptide	Protein	Low	Low	Low	No risk of virus	Requires extensive optimization, variable efficiency
		Gold nanoparticle	Protein	Medium	Low	Medium	No risk of virus	
							
	**Viral delivery methods**	Lentivirus	DNA	High	High	Low	Large cloning capacity	Random integration; insertional mutagenesis
						
		Adenovirus	DNA	Medium	Medium	Medium	Non-integrating	Immune response
						
		Adeno-associated virus	DNA	Medium	Low	Medium	Non-integrating	Limited cloning capacity
						
		Extracellular vesicle	Protein	Medium	Low	Low	Non-integrating; multiplexible; all-in-one format	Limited quantification method

***Therapeutic application of CRISPR-Cas system***

The CRISPR-Cas, as a great potential genome editing tool, has been employed in a wide range of therapeutic disorders, such as cancer^[^^118^^,^^119^^]^, as well as genetic, infectious and neurodegenerative diseases (Table 4). Hemoglobinopathies, such as β-thalassemia and SCD are a group of autosomal recessive hereditary single-gene diseases caused by absence/reduced β-globin chain synthesis or production of a structurally unstable β-globin chain in the hemoglobin tetramer. β-thalassemia, a prevalent monogenic and inherited blood disorder, is globally caused by more than 300 different point mutations and small Indels within the human *HBB* gene[^120^,^121^]. In recent years, the only available treatment for β-thalassemia and SCD has been allogeneic bone marrow transplantation. However, the limited availability of human leukocyte antigen-matched donors makes it a challenging and restricted approach. Thus, there is no defined therapy for people suffering from β-thalassemia and SCD. Alternatively, allogeneic HSC gene therapy, which relies on the transmission of the normal HBB via lentiviral vectors, is a promising approach for treating thalassemia and SCD^[^^122^^-^^124^^]^. The first cell-based gene therapy entitled Zynteglo (betibeglogene autotemcel), an autologous CD34^+^ cells encoding globin gene for the treatment of individuals with transfusions dependent β-thalassemia, was approved on August 17, 2022 by the US Food and Drug Administration (FDA)^[^^125^^,^^126^^]^. The discovery of CRISPR-Cas9 in 2012, which revolutionized the field of genetic manipulation, has emerged as a promising player in the gene therapy approach. Multiple research groups have used CRISPR-Cas9 in patient-derived HSPCs and patient-derived iPSCs to repair β-thalassemia and SCD mutations in the HBB^[^^124^^,^^126^^,^^127^^,^^130^^,^^154^^-^^158^^]^ Moreover, recent therapeutic strategies are based on the use of CRISPR- Cas9 to reactivate HbF expression. Interestingly, the results of studies have shown that increased HbF can mitigate the clinical symptoms of these diseases^[^^128^^,^^129^^-^^131^^,^^159^^,^^160^^]^. BCL11A is a master regulator of γ-globin gene that silences and inhibits HbF expression^162^. In previous studies, it has been demonstrated that targeted disruption of the BCL11A erythroid-specific enhancer using CRISPR-Cas9 in the second intron, increases the production of HbF, thereby ameliorating the severity of β-thalassemia and SCD, which can be considered a potential curative therapeutic strategy for β-hemoglobinopathies^[^^161^^-^^164^^]^. CTX001 is an ex vivo CRISPR-Cas9 gene-edited therapy for reactivating HbF in HSCs from patients suffering from SCD and transfusion-dependent β-thalassemia[^165^]. Several clinical trials are underway to assess the safety and efficacy of CRISPR system in β-hemoglobinopathies and other diseases (Table 5).

**Table 4:** Applications of CRISPR/Cas technology as therapeutic strategies

**Disease**	**Target genes**	**Editing method**	**Reference**
β-thalassemia	*HBB*	Deletion mutation	^[^^128^^]^
	*BCL11A*	Gene deletion	^[^^129^^,^^130^^]^
			
SCD	*HBB*	Insertion	^[^^131^^]^
	*BCL11A*	NHEJ/Indel	^[^^132^^]^
			
Hemophilia A	*F8*	Inversion-corrected iPSC cells	^[^^133^^]^
			
Hemophilia B	*F9*	Gene correction	^[^^134^^]^
			
HD	*HTT*	Deletion/NHEJ	^[^^135^^]^
			
Cystic fibrosis	*CFTR*	Gene correction	^[^^136^^]^
			
Duchenne musculardystrophy	*Dystrophin *	Gene correction/deletion	^[^^137^^-^^140^^]^
			
Diabetes mellitus type 1	*DMPKexon 15*	Gene editing	^[^^141^^]^
			
Cancer	*PD-1*	Gene knockout	^[^^142^^]^
			
HIV	*U3 LTR region*	Gene knockout	^[^^143^^]^
			
HIV	*CCR5 *	Gene knockout	^[^^144^^-^^146^^]^
			
HPV	*E6 and E7*	Gene knockout	^[^^147^^,^^148^^]^
			
HBV	*cccDNA*	Gene knockout	^[^^149^^-^^151^^]^
			
*Phlebotomus papatasi*	*rel*	Gene knockout	^[^^152^^]^
			
Malaria	*FREP1*	Gene knockout	^[^^153^^]^
			
Zika virus	*RNA*	RNA editing	^[^^154^^]^

*Duchenne muscular dystrophy*

DMD is a rare neuromuscular and X-linked recessive disease in children caused by mutations in the dystrophin gene and characterized by muscle weakness, loss of movement, and early death. DMD occurs about 1 in 3,500 to 5,000 newborn males worldwide. The most common type of mutation in the dystrophin gene involves the deletion of one or more exons, leading to frameshift or nonsense mutations, resulting in the absence of functional dystrophin protein[^166^-^168^]. In 2014, Long et al.^[^^136^^]^ used the CRISPR-Cas9 system for the first time in mouse zygotes to correct mutations and restore the expression of dystrophin. In 2017, Lattanzi et al.^[^^137^^]^ reported successful CRISPR-Cas9-mediated restoration of dystrophin expression in DMD myoblasts by removing the duplication of exon 2 and intron 2 in the dystrophin gene. In 2017, El Refaey et al.^[^^139^^]^ used the CRISPR-Cas9 technique for deletion of exon 23 in dystrophic mice in which the restoration of dystrophin protein was confirmed. In 2019, Min and Lee^[^^138^^]^ used adeno-associated virus serotype 9-mediated Cas9 and sgRNA to restore the exon 50 deletion and exclude exon 44 mutations in cardiomyocytes obtained from patient-derived iPSCs, as well as in a mouse model. The results showed the capability of CRISPR to remove mutations that result in DMD.

**Table 5:** Current clinical trials in hematology using the CRISPR/Cas9 system

**Diseases**	**Clinical trial no.**	**Method**	**Type of edit**	**Target gene**	**Phase**	**Delivery method**	**Sponsor**
β-thalassemia	NCT03655678	Cas9	Gene disruption	*BCL11A*	II/III	Electroporation (ex vivo)	Vertex Pharmaceuticals
							
HIV	NCT05144386	Cas9	Gene disruption	Three undisclosed genomic sites in the HIV DNA	II	AAV9 (in vivo)	Excision BioTherapeutics
							
Sickle cell disease	NCT03745287	Cas9	Gene disruption	*BCL11A*	II/III	Electroporation (ex vivo)	Vertex Pharmaceuticals
							
Acute lymphoblastic leukemia, T-ALL	NCT04984356	Cas9	Gene knock-out	*CD7*, *TRAC*	II	Electroporation (ex vivo)	Wugen inc.
							
AML	NCT05066165	Cas9	Gene knock-out, gene knock-in	WT1-specific TCR (knock-in), TRAC (knock-out), *TRBC* (knock-out)	II	Undisclosed (ex vivo)	Intellia Therapeutics
							
Metastatic gastrointestinal epithelial cancer, GI,	NCT04426669	Cas9	Gene knock-out	*CISH*	II	Ex vivo	Intima Bioscience, Inc.
							
Multiple Myeloma, MM,	NCT04244656	Cas9	Gene knock-out, gene insertion	BCMA	I	Undisclosed (ex vivo)	CRISPR Therapeutics AG
							
Non-small cell lung cancer	NCT05566223	Cas9	Gene knock-out	*CISH*	II	Ex vivo	Intima Bioscience, Inc.
							
Blindness, Leber Congenital Amaurosis,	NCT03872479	Cas9	Gene correction	*CEP290*	II	Adeno-associated virus (AAV5) (in vivo)	Editas Medicine, Inc.
							
Type 1 diabetes	NCT05565248	Cas9	Disruption, insertion	-	II	Ex vivo	CRISPR Therapeutics AG

CISH, cytokine inducible SH2 containing protein; BCMA, b-cell maturation antigen

*Cystic fibrosis*

CF is the most common autosomal recessive and monogenic lung disease that occurs due to mutations in the *CFTR* gene[^169^]. The CFTR protein is a chloride channel located on the surface membrane of epithelial cells and transports small ions through the membrane of multiple organs, including lung, intestine, and pancreas[^170^]. CRISPR-Cas system has been used to correct CFTR mutations in iPSCs from CF patients, as well as cultured intestinal stem cells. Researchers have utilized the CRISPR-Cas system to precisely repair CFTR-bearing homozygous F508 deletions (F508del) in exon 10[^171^^,^^172^]. 

*Huntington’s disease*

HD is a genetic disorder caused by a mutation in the *HTT* gene. The mutation leads to the production of an abnormal protein that accumulates in the brain, causing damage to nerve cells and leading to progressive neurological symptoms such as involuntary movements, cognitive decline, and psychiatric disturbances^[^^173^^]^. Currently, there is no cure for HD, and the available treatments only alleviate symptoms. CRISPR-Cas9 technology offers a promising approach for treating HD by correcting the underlying genetic mutation. CRISPR technology can be used to remove or replace the mutated section of the HTT gene with a healthy version. In one study, researchers used CRISPR-Cas9 to selectively target and edit the mutant HTT gene in mouse models of HD. The treatment resulted in significant improvements in motor function and reduced accumulation of mutant HTT protein in the brain^[^^174^^]^. Another study used CRISPR-Cas9 to correct the HTT gene mutation in human embryonic stem cells derived from patients with HD. The corrected cells were then differentiated into neurons, which showed normal levels of HTT protein expression and function[^175^]. While these studies show promise for using CRISPR-Cas9 as a treatment for HD, there are still challenges that are required to be addressed before it can be applied clinically. One major concern is ensuring that CRISPR-Cas9 targets only the mutant HTT gene and no other genes with similar sequences. Additionally, delivery methods are needed to be optimized to ensure the efficient delivery of CRISPR-Cas9 into target cells without causing off-target effects or immune reactions. While there are still challenges to overcome, continued research in this area could lead to a cure for this devastating disease.

*Blindness*

CRISPR technology has the potential to treat various genetic disorders, including inherited forms of blindness. IRDs are a group of genetic disorders that affect the retina, leading to vision loss and blindness. These diseases are caused by mutations in genes that are essential for the function and survival of photoreceptor cells in the retina^[^^142^^]^. CRISPR-Cas9 can be used to correct these mutations by targeting and editing the DNA sequence of the affected gene. Several studies have demonstrated the potential of CRISPR-Cas9 in treating IRDs. In 2017, researchers used CRISPR-Cas9 to correct a mutation in a gene called CEP290, which is associated with Leber congenital amaurosis, a severe form of childhood blindness. The study showed that CRISPR-Cas9 could effectively correct the mutation in human cells and restore normal protein function[^176^]. Researchers have used CRISPR-Cas9 to correct mutations in genes associated with retinitis pigmentosa, another form of inherited blindness that impairs vision due to the loss of photoreceptor cells, which can lead to irreversible blindness[^177^^,^^178^]. Recently, Tsai et al.[^179^] used CRISPR/Cas9 to remove and replace Rho. The authors tested this method on two mouse samples against the P23H and p.Asp190Asn mutations harboring the CRISPR/Cas system using the dual vector system AAV2/8 and the exogenous *RHO* gene. Both models showed an increase in outer nuclear layer thickness of up to 35% and a significantly improved ERG response after removal and replacement, in contrast to the addition of only one gene. The study showed that CRISPR-Cas9 could effectively correct these mutations in patient-derived cells and restore normal protein function. While these studies show promising results, there are still challenges needed to be addressed before CRISPR-Cas9 is used as a clinical treatment for IRDs. These include improving delivery methods for carrying CRISPR components to target cells within the retina and ensuring safety and efficacy in clinical trials.

***Infectious diseases***

*Human immunodeficiency virus*

AIDS, caused by HIV-1, is still a severe health problem worldwide. According to the World Health Organization (http://www.who.int/hiv/en/), 38.4 million people in the world were living with HIV in 2021, HIV-1 is an enveloped retrovirus comprising two copies of a 9.8 kb positive-sense RNA genome flanked by two LTR sequences. The HIV genome encodes proteins required for its life cycle, including gag, env, pol, vif, vpr, tat, vpu, rev, nef, and the antisense protein[^180^]. HIV-1 enters host cells by initial binding of its gp120 Env protein to the CD4 receptor on the surface of target cells, including CD4^+^ T helper lymphocytes, macrophages, and microglial cells. This interaction induces subsequent binding with the chemokine receptor CCR5 or CXCR4 as a co-receptor on the membrane of the target cell, which is essential for fusion between the virus Env and the cell membrane[^181^,^182^]. Highly active antiretroviral therapy is the primary method for controlling viral load by suppressing virus replication but fails to eradicate latent viral reservoirs in patients. In recent years, the CRISPR-Cas system has also provided new hope to cure HIV-1/AIDS. In 2014, Hu et al.^[^^142^^|^ used the CRISPR-Cas system to target LTR regions at both ends of viral genes in HIV-1 latent-affected myeloid lineage, promonocytic, and T cells with significant loss of LTR expression. In addition, blocking CCR5, an essential co-receptor for HIV-1 entry into the host cell, is considered the most potential therapeutic strategy for AIDS. In 2013, Cho et al.^[^^183^^]^ utilized the SpCas9 to induce mutations with a frequency ranging from 5% to 33% in human cells within the *CCR5* gene. In 2014, Mandal et al.[^145^] used the CRISPR-Cas9 genome editing tool to delete the *CCR5* gene in CD34^+^ HSPCs. Their results showed 42% biallelic inactivation frequency for *CCR5* in CD34^+^ HSPCs with low off-target mutagenesis. Another essential co-receptor that mediates HIV-1 entry into the cells is CXCR4. In 2015, Hou et al.[^184^] utilized the CRISPR-Cas9 genome editing system to disrupt the CXCR4 gene in human primary CD4^+^ T cells. The edited cells showed resistance to X4-type HIV-1 infection, as well as 40% mutagenesis with low off-target effects. Additionally, some research groups applied the CRISPR-Cas9 tool to investigate the simultaneous manipulation of *CXCR4* and CCR5 genes in various cell lines. The results showed that the manipulated cells were significantly protected from HIV-1 infection without off-target mutagenesis or cytotoxicity effect[^145^^,^^144^].

*Human papillomaviruses*

HPVs are double-stranded DNA viruses with a genome size of about 8000 bp. More than 200 types of HPVs have already been identified. HPV is the major cause of cervical cancer in women, as well as anogenital and head and neck cancers in men. More than 50% of HPV-positive cervical cancers are associated with HPV16, and about 12% with HPV18, which are both considered high-risk HPVs[^185^^-^^188^]. HPV E6 and E7 are the vital oncogenes responsible for accelerating HPV-induced carcinogenesis[^188^]. CRISPR-Cas9 has been used to target the HPV16 and HPV18 E6 and E7 in HeLa cells, as well as cervical carcinoma cell lines, which results in the induction of p53 or Rb, leading to cell cycle arrest and cancer cell death[^189^]. In 2014, Zhen et al.^[^^190^^]^ targeted the promoter of HPV16 E6 and E7 using the CRISPR-Cas9 in SiHa cells and tumor animal models. Their results showed the induction of p53 and p21 proteins, which resulted in the inhibition of tumorigenesis.

*Hepatitis B virus*

Hepatitis B is a contagious liver infection caused by the HBV, a major global public health problem. Many antiviral agents have been used to cure HBV infection; however, they are not effective in eradicating HBV due to their inability to remove cccDNA, a template used by HBV for replication. cccDNA completely persists during therapy and cannot be eliminated by current antiviral therapies[^191^]. Thus, new treatments are required to effectively eliminate the cccDNA. Many research groups have used CRISPR-Cas9 to target the HBV core and surface proteins in animal models and hepatoma cell lines such as, HepG2, Huh-7, HepG2-H1, and HepG2.2.15. The results showed that the elimination of episomal cccDNA led to the inhibition of HBV replication and expression both in vitro and in vivo[^148^^-^^150^,^192^].

**Fig. 3:**
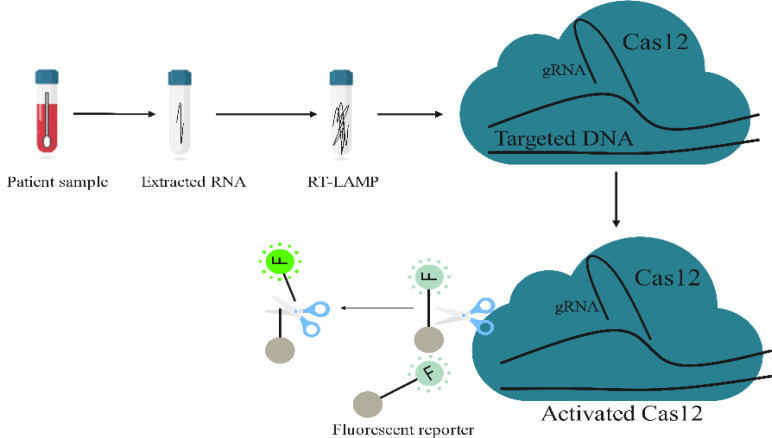
The general principle of nucleic acid detection by the DETECTR based on CRISPR/Cas 12. Nucleic acid is extracted from clinical samples, and the target sites are amplified by RT-RPA. Detection is performed in a reaction mixture containing T7 RNA polymerase, Cas12a, a target-specific gRNA, and an ssDNA probe that fluorescence when cleaved. Gray circles indicate quenchers that are connected by a short oligonucleotide. F, FAM

*Severe Acute Respiratory Syndrome Coronavirus 2*

In December 2019, a novel beta coronavirus, namely SARS-CoV-2, was identified in Wuhan, China. SARS-CoV-2 causes a respiratory illness called COVID-19. The SARS-CoV-2 virus quickly spread worldwide and led to a pandemic[^193^,^194^]. A low-cost, rapid, simple, and sensitive point-of-care testing for the early detection of viruses in pandemics such as COVID-19 was required. In this regard, the CRISPR genome editing system can be applied for nucleic acid detection, owing to its exclusive features. For this purpose, researchers have utilized different types of CRISPR for the design and development of CRISPR-based diagnostic systems. Broughton and colleagues^[^^195^^]^ used the called DETECTR. In CRISPR-Cas12a to develop an accurate, simple, and rapid (<40 min) diagnostic method for SARS-CoV2 this method, gRNAs were designed to detect SARS-CoV-2, bat SARS-like coronavirus, and SARS-CoV in the E gene and specifically detects only SARS-CoV-2 in the N gene (Fig. 3). Ding et al.^[^^196^^]^ enveloped an all-in-one dual CRISPR-Cas12a diagnostic method rapidly targeted nucleic acids. In this method, all components for CRISPR-mediated detection and nucleic acid amplification are merged into a single reaction. CREST was introduced by Rauch et al.^[^^197^^]^ for SARS-CoV-2 detection. The CREST assay was developed to address the main barriers of virus detection, including high cost, limited access to equipment and materials, and lack of highly trained operators. Azhar et al.^[^^198^^]^ applied a Cas9 ortholog from FnCas9 to develop FELUDA. In the FELUDA-based lateral flow assay, gRNAs were designed to target the NSP8 and nucleocapsid phosphoprotein of SARS-CoV-2 with rapid, cost-effective, and machine-independent detection of a small amount of SARS-CoV-2 RNA within one hour. Recently, Yoshimi et al.^[^^199^^]^ have developed a CRISPR/Cas3-based detection method called CONAN. The CANON assay combines isothermal amplification methods for a fast, sensitive, and tool-free diagnosis of SARS-CoV-2.

***Parasites***

Parasites are biological organisms that live and feed on another organism, known as a host. These organisms can cause severe health issues in humans and animals, making the development of new treatments highly necessary. In principle, parasitic diseases are the main reason for morbidity and economic constraints. CRISPR gene editing technology has emerged as a powerful tool in the fight against parasites. With CRISPR, genetic sequences can be specifically targeted and edited to make precise modifications. CRISPR/Cas9-mediated gene editing, for instance, could be used to disrupt the reproduction of parasites or selectively edit their virulence genes, which would lead to the reduced parasite transmission and virulence, ultimately aiding in the development of novel methods for controlling parasitic infections. Moreover, CRISPR has been used to study the functional genomics of parasites, offering new insights into their biology and identifying potential drug targets. A study demonstrated the use of CRISPR/Cas9-mediated gene editing to investigate new targets for drug development against the protozoan parasite Trypanosoma brucei, which causes African trypanosomiasis. The study identified several proteins that play an essential role in the parasite's life cycle, which could be targeted with new therapeutics^[^^200^^]^. Additionally, CRISPR technology could potentially be used to develop vaccines against parasites by enabling the manipulation of antigens to enhance host’s immunity. CRISPR technology also offers the possibility of creating genetically modified parasites that can serve as live vaccines[^201^]. Hence, the design and development of proper detection methods and efficient treatments are essential. CRISPR-Cas9 has been used for genome editing of *Leishmania*, *Plasmodium* spp., *Toxoplasma gondii*, and *Trypanosoma cruzi*. It has also been employed in the modification of medically important vectors such as different species of mosquitos and water fleas. Understanding diverse aspects of parasite biology has greatly benefited from the development of CRISPR technology. Malaria is a life-threatening and severe disease caused by the *Plasmodium* parasites species, which is transmitted to humans through the bite of infected female mosquitoes of the Anopheles genus. Malaria remains a public health threat worldwide, causing the death of more than 400,000 people annually[^202^^,^^203^]. Genome editing technology, especially the CRISPR/Cas9 system, enables researchers to investigate genetically engineered mosquitoes to eradicate malaria. In 2014, Ghorbal et al.^[^^204^^]^ successfully used the CRISPR/Cas system to edit the genome of *P. falciparum* through gene disruption and single-nucleotide gene editing. In addition, they generated an artemisinin-resistant strain by introducing a mutation (C580Y) in the PF3D7_1343700 K13-propeller. Artemisinin-based combination therapy is a primary treatment for *P. falciparum*, and mutations in K13-propeller domains are associated with the artemisinin phenotype. The sexual phase of malaria parasites, which results in the formation of infectious sporozoites, is necessary to be completed by transmission to a mosquito vector. Inhibiting the fertility of mosquitoes through chemical spraying or genetically modifying the population to render them infertile is one of the most effective strategies to combat malaria. Transgenic mosquitoes do not transmit the malaria parasite because they express inhibitors of Plasmodium growth. Gantz et al.[205] used the CRISPR/Cas9 system to integrate antiparasite effector genes against the *P. falciparum* ookinete proteins, such as single-chain antibodies m1C3 to target chitinase 1 and m2A10 to target CSP, to prevent malaria transmission. In 2018, Dong et al.^[^^152^^]^ used the CRISPR/Cas9 system to eliminate the *FREP1* gene, which encodes an immune protein, from the genome of *Anopheles gambiae* mosquitoes, to generate mosquitoes that are highly resistant to malaria parasites. *FREP1* acts as a receptor for plasmodium on the midgut of the Anopheles mosquito. Consequently, the knockout of the *FREP1* mosquitoes makes them resistant to the parasite and significantly reduces the likelihood of transmitting the malaria parasite to the host. The CRISPR system has widely been used to knock out target genes, replace nucleotides, and tag endogenous loci with epitope tags in order to permanently alter the target genes. The CRISPR tool, however, has only been sporadically used to control gene expression through epigenetic regulation. A dCas9 was successfully used in a recent study by Xiao et al.^[^^206^^]^ to epigenetically regulate the transcription of genes related to parasite invasion, including reticulocyte binding protein homology 4 (RH4) and erythrocyte binding antigen 175. In order to activate the expression of RH4, the GCN5, as a histone acetyl transferase, was fused to the dCas9 (dCas9GCN5). The recombinant dCas9GCN5 was specifically directed to the promoter region of the *rh4* gene by the RH4sgRNA. The findings showed a significant overexpression of the *rh4* gene in *P. falciparum* using the CRISPR/dCas9GCN5 system. Moreover, PfSir2a, as a histone deacetylase, was fused to dCas9 (dCas9Sir2a) to repress the expression of the *eba-175* gene in *P. falciparum*. The *P. falciparum* *eba-175* gene was disrupted using CRISPR/dCas9Sir2a, which inhibits parasites from invading erythrocytes treated with chymotrypsin. Furthermore, the gene PfSET1 of *P. falciparum*, which is essential for the growth of its *P. falciparum *asexual stage, was suppressed using the CRISPR/dCas9Sir2a system. The phenotypic changes in parasite growth were caused by the precise inactivation of the PfSET1 gene. In addition to the asexual blood stages of the malaria parasite life cycle, the CRISPR tool has been applied to identify the functional role of genes in other steps of the parasite's life cycle. Both eukaryotic organisms and malaria parasites use many of the same transcription factors. Unexpectedly, a family of genes encoding Apetala2 (ApiAP2) proteins, which are essential for parasite development in Plasmodium, has been discovered in many plants. In a study performed by Zhang et al.[207], 12 out of 26 *Plasmodium yoelii ApiAP2* genes were successfully knocked out using the CRISPR/Cas9 system, and the findings revealed the crucial role of some of the genes in the development of gametocytes, oocysts, and sporozoites. The CRISPR/Cas9 system is also used to identify antimalarial compounds and elucidate the mechanisms underlying their actions. Benzoxaboroles have demonstrated strong activity against a variety of infectious pathogens. Using the CRISPR/Cas9 system, Sonoiki et al.[^208^] demonstrated the potent anti-malarial activity of AN3661 (a benzoxaborole) against cultured *P. falciparum* asexual blood stage parasites. They made mutations to the active site of the cleavage and polyadenylation specificity factor subunit 3 (CPSF3) gene where AN3661 should bind. In addition to malaria, CRISPR technology could be used to create genetically engineered mosquitoes that are resistant to infection by the Zika virus. The Zika virus is a flavivirus spread by the bite of infected mosquitoes of the *Aedes aegypti* species, which causes fever, rash, red eyes, muscle pain, headache, and birth defects and is linked to the Guillain-Barré syndrome. There is no specific treatment and vaccine for the Zika virus[^209^]. In 2022, Chen et al.^[^^153^^]^ used CRISPR-Cas13b to develop an anti-Zika system in 293T cell lines. The study demonstrated the efficacy of CRISPR-Cas13b as an anti-RNA virus therapy by degrading targeted viral RNA with Cas13. Leishmaniasis is a neglected tropical disease caused by protozoan parasites from over 20 *Leishmania *species. This vector-borne disease is transmitted by about 30 species of infected phlebotomine sandflies and is widespread in 97 countries throughout the world. In spite of comprehensive research on various aspects of this infectious disease, there is currently no effective or preventive strategy against leishmaniasis^[^^210^^]^. In 2015, Sollelis et al.^[^^211^^]^ applied the CRISPR-Cas9 system to knock out the paraflagellar rod-2 genes (a tandemly repeated gene family) in the *Leishmania major* parasite. As a result, they obtained null mutants in a single round of transfection, as well as the absence of off-target editions. In 2015, Zhang et al.^[^^212^^]^ developed two loss-of-function Indel mutations in *Leishmania donovani* through the precise deletion of the 3-kb from *LdMT* gene using CRISPR-Cas9. Furthermore, they identified a novel single-point mutation created by CRISPR-Cas9 in the *LdMT* (M381T) gene that led to miltefosine resistance, a concern for the only available oral antileishmanial drugs. Several research groups have successfully removed genes, such as Cysteine peptidases, LeishIF4E-3, LeishIF4E1, ubiquitin, RAD51-related genes, flagellar motility factor Pf16, adenine phosphoribosyltransferase, casein kinase 1.1, and HSP23, along with HSP100, from various Leishmania species, including *Leishmania mexicana*, *L. major*, *Leishmania tarentolae*, *L. donovani*, and *Leishmania braziliensis*[^213^-^218^]. Chagas disease is an inflammatory and infectious disease caused by *Trypanosoma cruzi*. Peng et al.^[^^219^^]^ used CRISPR-Cas9 in *T. cruzi* to knock down expression of β-galactofuranosyltransferase enzyme, which plays a role in *T. cruzi *pathogenesis. The results showed a significant downregulation in enzyme production without evident off-target mutations. In another study, Lander et al.^[^^220^^]^ used CRISPR-Cas9 to improve the analysis of the functionality of the *T. cruzi *genome. In this regard, sgRNAs were designed to disrupt the expression of GP72-related genes and paraflagellar rod proteins 1 (PFR1) and 2 (PFR2). The results showed that the PFR1, PFR2, and GP72 proteins contribute to the attachment of flagellar to the cell body and also to the motility of the parasites. Scientists are also utilizing the CRISPR-Cas9 system for gene editing in mosquitoes to modify their genes, with the aim of inhibiting the parasite's life cycle and reducing their ability to spread pathogens[^221^,^222^]. CRISPR can be used to modify a wide range of parasites, including Leishmania, Toxoplasma, Trypanosoma, and Plasmodium. For instance, the Cas9/T7-RNAP construct can be used to induce gene deletion or disruption mutants in Leishmania species. This technique can also be utilized to develop gene drive strategies in insect vectors that transmit parasites from one host to another, which could potentially help reduce the transmission of diseases caused by these parasites. In addition, active RNA interference machinery has been developed for use in parasitology research, which can be employed to knockdown or knock-in target gene into various parasite systems, such as Trypanosoma and Plasmodium. This technology enables gene deletions or insertions with high transgene copy numbers and compensatory adaptations that enable the parasite to survive and replicate even after the targeted gene is deleted or disrupted. CRISPR has also been explored as a potential tool for creating gene drive strategies in other systems, such as Trypanosoma. Such strategies could potentially increase transgene copy numbers while reducing vector populations through increased mortality rates of the infected individuals or reduced ability of the infected individuals to transmit the disease agent^[^^205^^]^.

*CRISPR-based diagnostic methods for detection of parasites*

The CRISPR system has been applied for parasite detection. In recent years, accurate parasite diagnosis has been made possible by combining parasitological and CRISPR/Cas systems. In 2022, Dueñas et al.^[^^223^^]^ developed a CRISPR-based diagnostic tool using the CRISPR-Cas12a system to detect *Leishmania* spp. Two gRNAs were designed to target the 18S rDNA (18S ribosomal RNA gene), a highly conserved region across Leishmania species, and kDNA minicircles that are conserved in the *Leishmania* (*Viannia*) subgenus. They could identify 5 × 10^0^ (18S rDNA) and 5 × 10^2^ (kDNA) parasites/reactions using this system[^224^]. CRISPR-based malaria screening has been emerged as a promising method for detecting the presence of malaria parasites in blood samples. This method utilizes the CRISPR/Cas system to target and cleave specific sequences of the parasite genome present in a blood sample, which can then be detected using various molecular methods, such as PCR or fluorescence assays. One of the main advantages of using CRISPR-based malaria screening is its high specificity and sensitivity. Additionally, it has the potential to greatly improve diagnostic accuracy and speed, compared to the traditional methods such as microscopy. Furthermore, CRISPR-based malaria screening can also be used to detect drug-resistant strains of the parasite by targeting specific mutations in the genome associated with resistance. This technology has immense potential in preventing and controlling the spread of malaria, especially in regions with limited access to the advanced diagnostic resources. In a study, researchers utilized the CRISPR/Cpf1 (Cas12a), a SHERLOCK-based diagnostic platform, to establish an ultrasensitive, one-pot, lyophilized, and free nucleic acid extraction method for detecting and distinguishing *P. falciparum *(Pfr364 gene), *Plasmodium vivax *(18s rRNA), *Plasmodium ovale *(18s rRNA), and *Plasmodium malariae *(18s rRNA). The results indicated an ultrasensitive and specific assay for differentiating *P.* *falciparum* and *P. vivax* in clinical samples and were able to detect fewer than two parasites per microliter of blood[^225^]. Overall, CRISPR-based malaria screening represents a promising approach for improving malaria diagnosis and management, with potential applications in both clinical settings and field studies. In the future, further research will be necessary to optimize CRISPR-based malaria screening technology and expand its potential applications.

*Ethical implications of fighting malaria with genome editing*

CRISPR-Cas9 technology has shown promising results in the development of novel strategies for combating parasitic infections and controlling mosquito populations, which are important vectors for many parasitic diseases. CRISPR has been used to genetically modify mosquitoes so that they are unable to transmit diseases, such as malaria and dengue fever. CRISPR has also been utilized to study the function of genes in parasitic organisms and understand their biology, which can lead to the development of new drugs and therapies for parasitic infections affecting millions of people worldwide[^226^]. While the potential use of genome editing technology to combat malaria is promising, it raises ethical concerns that need to be addressed. Some of the concerns in using genome editing technology to combat malaria include safety-related issues, equity, and the unintended consequences of altering the mosquito population along with potential environmental impacts. When considering the implementation of genome editing technology for malaria control, it is of most importance to thoroughly evaluate and weigh the potential risks and benefits. This evaluation should not only include ethical concerns but also take into account the views of local communities and stakeholders who would be impacted by such interventions. Overall, the use of CRISPR technology in parasitology and mosquito control holds great promise for improving global public health and reducing the burden of parasitic infections and mosquito-borne diseases. Continued research and development in this field will be critical to fully realize the potential of CRISPR technology in addressing some of the most pressing public health challenges worldwide. It is important for scientists, policymakers, and stakeholders to engage in thoughtful discussions and collaborations to ensure that the use of this technology is safe, ethical, and effective. Furthermore, it will be crucial to ensure that these advances are accessible and affordable for those who live in low-resource countries where parasitic infections and mosquito-borne diseases are highly prevalent. In conclusion, while there are still challenges and uncertainties to be addressed using CRISPR technology for parasitology and mosquito control, it has the potential to revolutionize our approach to global health issues and improve the lives of millions of people around the world. 

***CRISPR protein tagging***

CRISPR protein tagging is a revolutionary technique that allows for precise labeling and visualization of proteins in living cells. This method involves using CRISPR-Cas9 to introduce a DNA construct that contains the protein of interest fused with a fluorescent tag or affinity tag. The tagged protein can then be tracked and studied in real-time, enabling researchers to gain a better understanding of its function within the cell. This technique has the potential to revolutionize our understanding of protein function and localization, providing more precise analysis and manipulation of cellular processes. In the field of parasitology, CRISPR protein tagging has proven to be particularly useful in studying the complex lifecycle and host interactions of parasites. The precise tagging of proteins within parasites has been a challenging task for many years. This technology has already led to important discoveries about the mechanisms underlying malaria transmission and infection and has the potential to uncover new targets for drug development. Additionally, CRISPR protein tagging could be used to explore the diversity of parasitic species and their adaptations to different environments, providing insight into the evolutionary processes that have shaped these organisms over time. Overall, this cutting-edge technique is poised to significantly advance our understanding of parasitic diseases and improve our ability to combat them. One of the key advantages of CRISPR protein tagging is its versatility. This technique can be applied to a wide range of parasitic organisms, from single-celled protozoa to complex multicellular worms. It also allows researchers to study different stages in the life cycle of parasites, such as the transmission from vector to host or the development within specific organs. By labeling different proteins within these stages, scientists can gain a better understanding of how parasites interact with their environment and host cells. Furthermore, CRISPR protein tagging can be used in combination with other techniques, such as transcriptomics and proteomics, to generate comprehensive datasets that shed light on various aspects of parasite biology. As this technology continues to improve and become more widely adopted, we can anticipate even more exciting discoveries about parasitic diseases in the years ahead. Another advantage of CRISPR protein tagging is its potential for drug discovery. By identifying key proteins involved in virulence and drug resistance, researchers can develop targeted therapies that disrupt the parasite's ability to survive within the host. This approach has been proven to be successful in treating malaria. Clinical trials of drugs targeting specific enzymes have shown promises. CRISPR protein tagging can be used to screen large libraries of compounds for their ability to inhibit or enhance specific protein functions, potentially leading to the discovery of new drugs with novel mechanisms of action. Overall, this technology has the potential to significantly improve our ability to treat parasitic diseases and reduce their impact on global health[^227^,^228^]. Researchers have utilized CRISPR protein tagging to study PlsoT1, a protein found in the malaria parasite that is responsible for the invasion of host red blood cells. PlsoT1 is a surface protein that plays a crucial role in the initial stages of erythrocyte invasion by the malaria parasite. The ability to track PlsoT1 using CRISPR protein tagging has led to a better understanding of its localization and function during the invasion process. In addition, CRISPR protein tagging has been used to study other important proteins involved in malaria pathogenesis, such as PfEMP1 and CSP. With continued advancements in this technology, we can expect even more breakthroughs in our understanding of parasitic diseases and the development of new treatments. PfEMP1 protein is essential for the cytoadherence of *P. falciparum*-infected erythrocytes within the host, and its expression is associated with severe malaria. CSP protein is critical for the development of *P. falciparum* sporozoites, which are responsible for transmitting malaria from mosquitoes to humans. By using CRISPR protein tagging to track the location and activity of PfEMP1 and CSP proteins, researchers have gained a better understanding of how these proteins contribute to pathogenesis and sporozoite development and identify potential targets for drug development. Moreover, this technique has allowed for visualization of PfEMP1 and CSP trafficking within the infected cells, providing insights into the cellular mechanisms underlying cytoadherence and sporozoite formation[^227^]. CRISPR/Cas9-mediated endogenous C-terminal tagging has been utilized to study *T. cruzi* genes, specifically the IP3R. By tagging the IP3R protein and observing its localization within the cell, researchers have gained a better understanding of its role in acidocalcisome function. Acidocalcisomes are acidic organelles that store calcium and other essential ions in *T. cruzi*, making them an attractive target for drug development. The use of CRISPR/Cas9 technology has provided a more precise and efficient tagging proteins in *T. cruzi*, enabling investigators to evaluate the localization and function of proteins with greater accuracy. This technique has opened up new avenues for research on *T. cruzi* biology and potential therapies for Chagas disease[^228^]. CRISPR protein tagging has been used to investigate the role of DNA polymerases in *Trypanosoma brucei*, the parasite responsible for African sleeping sickness. By labeling these enzymes with fluorescent tags, scientists could track their movement and activity during different stages of the cell cycle, which provides valuable insights into the regulation of DNA replication in these parasites and leads to the discovery of potential targets for drug development. Similarly, CRISPR protein tagging has been employed to study RNA polymerases in *P. falciparum*. By understanding how these enzymes function within the context of parasite biology, we can develop new strategies to disrupt their activity and combat parasitic diseases. However, there are also some challenges associated with CRISPR protein tagging in parasitic organisms. One of the main challenges is the delivery of the CRISPR system into these organisms, which can be difficult due to their complex life cycles and cellular structures. In addition, off-target effects can occur when using this technique, potentially leading to unintended changes in gene expression or function. To mitigate these issues, researchers are required to carefully design and optimize their experiments to minimize off-target effects and ensure accurate labeling of target proteins.

## Future perspective and considerations

Until date, the CRISPR-Cas technology is the most potent gene editing tool and has been applied in various fields because of its simplicity and efficient gene-editing capability compared to previous gene editing tools. Although the CRISPR-Cas system has potential advantages in gene manipulation, concerns remain regarding off-target effects in which editing events happen at the untargeted regions within the genome. Therefore, investigations are underway to improve CRISPR-Cas activity and reduce off-target effects by optimizing the design of gRNA and applying different types of Cas or Cas9 variants. Despite the fact that off-target mutations on the genome could have unexpected effects on the parasite phenotype, this might not be a significant issue since apicomplexans have small genomes with less potential for off-target mutation. No off-target mutations introduced by Cas9 was reported in both *P.* *falciparum* and *P. yoelii*[^207^], indicating that this system is extremely specific in these parasites that lack NHEJ. However, it is advised to be further investigated in other apicomplexan parasites, such as *T. gondii*, which has a functioning NHEJ system and may also repair DSBs in off-target locations. Another issue related to the CRISPR-Cas system is immunogenicity, in which the immune system of the host cell will respond to Cas protein. A study recently underlined the question of whether immunological responses to Cas9 negatively impact its clinical use^[^^229^^]^. Anti-Cas9 responses have been detected in healthy human adults. The presence of anti-Cas9 antibodies does not necessarily indicate an immune response against Cas9-mediated gene editing; however, anti-Cas9 T cells are detected in the blood of test subjects. These T cells can react to Cas9 in circulation, as they are effectively presented Cas9 through major histocompatibility complex molecules. The CRISPR-Cas9 system may become ineffective if the immune system destroys the corrected cells. To minimize the risk of developing anti-Cas9 CTLs in CRISPR-Cas9 gene editing, known strategies should be employed. The potential of gene editing is promising, but we must be cautious in our approach. Gene therapy offers valuable insights, as it has found ways to overcome anti-capsid and anti-transgene CTL responses. This goal was achieved by carefully considering factors such as the vector, dose, target tissue, administration route, promoter, and immune suppression. By taking these factors into account, the gene-editing field can proceed with more confidence and ensure that it is being conducted safely and effectively. CRISPR-Cas9 platforms, leading to short-term expression of Cas9 should be developed for CRISPR-Cas9 platforms. Gene therapy has shown potential difficulties due to immune responses. Studies involving AAV-CRISPR-Cas9, which silences T cells, impairs product-specific CD8^+^ T cells and uses immune evasion by muscle-specific gene expression by muscle-specific gene expression, demonstrate potential difficulties. Results suggest that immune response may impact the efficacy of gene therapy and lead to the development of antibodies that can interfere with the treatment. Moreover, CRISPR-Cas9 can induce a p53-mediated DNA damage response, affecting the safety of gene therapy[^190^]. It is important to evaluate immune responses in gene editing in order to determine the most appropriate treatment approach and minimize the potential risks. There are several ways to address the issue of immune recognition. Some possible strategies consist of modifying the structure of Cas9 proteins to hide immunogenic epitopes, utilizing Cas9 orthologs from nonpathogenic bacteria, inducing immune tolerance or immune suppression, or focusing on immune-privileged organs, such as the eye. Additionally, altering antigen presentation of Cas9 epitopes is a promising approach derived from Epstein-Barr Virus, which interferes with proteasomal degradation and the subsequent antigen presentation[^230^]. While there is evidence suggesting that CRISPR-Cas systems can be immunogenic, further research is necessary to fully comprehend the potential risks and develop effective mitigation strategies. As the use of the CRISPR system continues to expand in various fields, it is crucial to consider ethical issues tha may arise carefully. Multiple improvements are necessary to address all current challenges related to the application of CRISPR in order to maximize on-target efficiency and minimize untargeted editing. 

In conclusion, the application of CRISPR technology in manipulating Leishmania and *T. gondii* genomes offers a promising approach to develop new treatments for these diseases. The use of CRISPR has enabled researchers to identify essential genes that are required for survival of the parasite, which can be targeted for drug development. Moreover, CRISPR can be used to study the biology of the parasite and provides insights into the mechanisms of pathogenesis and drug resistance. The potential of CRISPR in the field of parasitology is vast, and it is exciting to see how this technology will continue to revolutionize the field of genetic engineering in the future.

## DECLARATIONS

### Ethical statement

Not applicable.

### Data availability

Data supporting this article are included within the article.

### Author contributions

SE: conceptualization of research, design of study, analysis of data and interpretation, and preparation of manuscript; MAK: design of study, analysis of data and interpretation, and preparation of manuscript; AR: preparation of manuscript; MK: design of study, review and editing of the final manuscript, and validation and visualization; PP: design of study and validation and visualization. All authors reviewed and approved the final version for publication.

### Conflict of interest

None declared.

### Funding/support

This study presents partial outcomes of Ph.D. thesis written by Sahar Ebrahimi and granted by the Pasteur Institute of Iran (Tehran). The funders had no role in the study design, data collection and analysis, decision to publish or preparation of the manuscript.
